# Seroprevalence study in humans and molecular detection in *Rhipicephalus sanguineus* ticks of severe fever with thrombocytopenia syndrome virus in Thailand

**DOI:** 10.1038/s41598-024-64242-x

**Published:** 2024-06-11

**Authors:** Paola Mariela Saba Villarroel, Tanawat Chaiphongpachara, Elif Nurtop, Sedthapong Laojun, Tassanee Pangpoo-nga, Thanaphon Songhong, Dolruethai Supungul, Cécile Baronti, Laurence Thirion, Pornsawan Leaungwutiwong, Xavier de Lamballerie, Dorothée Missé, Sineewanlaya Wichit

**Affiliations:** 1 Department of Clinical Microbiology and Applied Technology, Faculty of Medical Technology, Mahidol University, Nakhon Pathom, Thailand; 2 Viral Vector Joint Unit and Joint Laboratory, Mahidol University, Nakhon Pathom, Thailand; 3 Department of Public Health and Health Promotion, College of Allied Health Sciences, Suan Sunandha Rajabhat University, Samut Songkhram, Thailand; 4 Unité des Virus Émergents (UVE: Aix-Marseille Univ-IRD 190- Inserm 1207), Marseille, France; 5 Phon Phisai Hospital, Phon Phisai District, Nong Khai Thailand; 6 Department of Microbiology and Immunology, Faculty of Tropical Medicine, Mahidol University, Bangkok, Thailand; 7 MIVEGEC, CNRS, IRD, Univ. Montpellier, Montpellier, France

**Keywords:** SFTSV, *Rhipicephalus sanguineus*, Thailand, Seroprevalence, Tick-borne diseases, Viral epidemiology, Infectious-disease diagnostics

## Abstract

Severe fever with thrombocytopenia syndrome virus (SFTSV) is an emerging tick-borne virus with a mortality rate of up to 30%. First identified in China in 2009, it was later reported in other Asian countries, including Thailand in 2020. SFTSV has been detected in several tick species, including *Rhipicephalus sanguineus*, known for infesting dogs. We conducted a seroprevalence study of SFTSV in Bangkok and Nong Khai, Thailand, by analyzing 1162 human samples collected between 2019 and 2023. The testing method relied on IgG detection using ELISA and confirmed though a virus seroneutralization test. The results indicated that out of the participants, 12 (1.1%) tested positive for anti-SFTSV IgG antibodies; however, none exhibited positive results in the seroneutralization assay. Additionally, molecular detection of SFTSV, Crimean-Congo hemorrhagic fever (CCHF), *Coxiella* spp., *Bartonella* spp., and *Rickettsia* spp. was performed on 433 *Rh. sanguineus* ticks collected from 49 dogs in 2023 in Chachoengsao Province, Thailand. No evidence of these pathogens was found in ticks. These findings highlight the importance of exploring viral cross-reactivity. Furthermore, it is important to conduct additional studies to isolate SFTSV from animals and ticks in order to identify the potential transmission routes contributing to human and animal infections in Thailand.

## Introduction

Severe fever with thrombocytopenia syndrome (SFTS), is an emerging tickborne disease caused by the severe fever with thrombocytopenia syndrome virus (SFTSV), also known as Dabie bandavirus (*Phenuiviridae* family, *Bandavirus* genus)^1–3^. SFTSV has been detected in a range of animal species including sheep, cattle, dogs, cats, deer, wild boards, raccoons, rodents, and hedgehogs^4^. Humans are incidental hosts who contract the virus through bites from SFTSV-carrying ticks, by direct contact with body fluids of infected patients or infected pets^2,5^*.* Clinical manifestations are broad and include fever, leukopenia (< 4000/mm^3^), thrombocytopenia (< 100,000/mm^3^), gastrointestinal symptoms, neurological symptoms, and multiple organ failure^3,6^, with a case-fatality rate ranging from 6 to 30%^3^.

*Haemaphysalis longicornis* commonly known as the Asian longhorned tick, acts as the primary transmission vector and reservoir, and is endemic in the Asia–Pacific region^2,3^. However, SFTSV has also been detected in several other tick species, including *Amblyomma testudinarium*, *Ixodes nipponensis*, and *Rhipicephalus microplus*^7^*.* Another tick species, *Rhipicephalus sanguineus* (Acari: Ixodidae), commonly referred to as the “brown dog tick”^8,9^ has also been demonstrated to play a potential vector role in SFTSV transmission^10^. It is the most common tick species that parasitizes dogs in both rural and urban areas worldwide, particularly in tropical and subtropical regions where its prevalence persists throughout the year. Occasionally, it infects humans, often associated with heavily infected dogs and highly infested environments^11^.

Severe fever with thrombocytopenia syndrome virus was first reported in humans in China in 2009, in rural areas of the Hubei and Henan provinces. It subsequently spread to other provinces, leading to outbreaks with thousands of cases. Later, SFTSV was identified in other Asian countries, including the Republic of Korea, Japan, Vietnam, Myanmar, Taiwan, and Thailand^5,12^. In Thailand, the virus was initially reported in 2020 and, as of January 2024, four cases have been identified. The first case was a 70-year-old woman with suspected transmission through cats^13^, and the remaining cases were identified in a retrospective study conducted among patients hospitalized in or near Bangkok between 2019 and 2020^14^.

Seroprevalence studies in humans have been conducted in China, Japan, Korea^3^, Pakistan^15^, and Vietnam^16^. In Thailand, seroprevalence studies in humans have not been performed, but a seroprevalence study in dogs was conducted in 2022, which demonstrated the presence of antibodies. In addition, the virus was also isolated from a dog^4^.

In the present study, we conducted the first human seroprevalence study in Thailand by analyzing serum samples from healthy individuals in Bangkok and from patients with dengue-like symptoms collected in Nong Khai Province to provide information on the circulation of SFTSV in the country. Furthermore, we performed molecular detection assays to detect SFTSV and other pathogens, including Crimean-Congo hemorrhagic fever (CCHF), *Coxiella* spp., *Bartonella* spp., and *Rickettsia* spp. in *Rh. sanguineus* ticks collected from dogs residing in rural communities of Tha Takiap Subdistrict, Chachoengsao Province, Thailand, to identify potential pathogen transmission by this tick.

## Materials and methods

### Human sample collection and ethical approval

**Figure 1:**
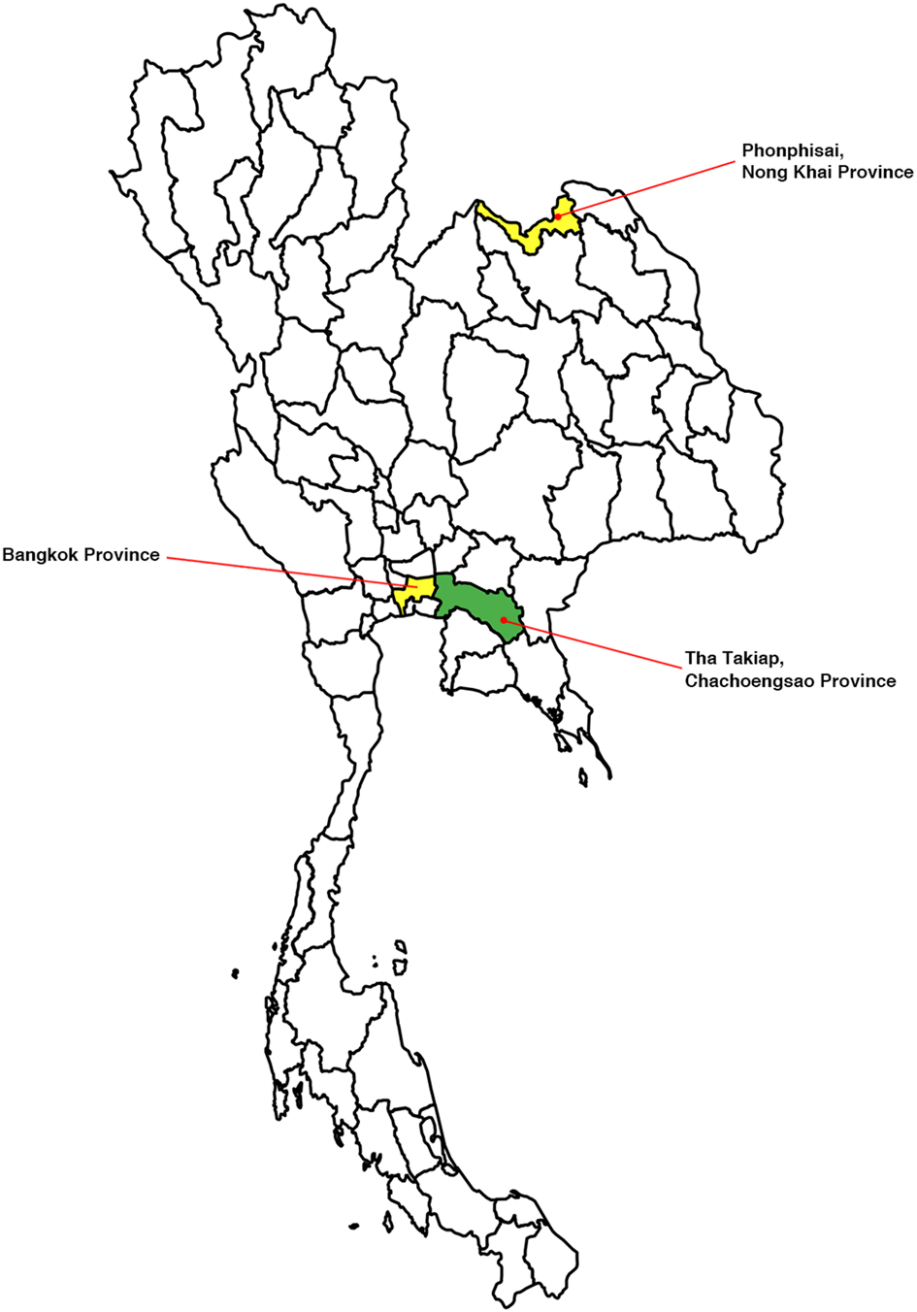
Geographical distribution map of sample collection sites. In yellow: human samples. In green: *Rhipicephalus sanguineus* ticks collected from dogs. The map was created using Mapchart (https://www.mapchart.net/).

One milliliter of residual serum samples was collected from 940 asymptomatic adult volunteer blood donors who attended the National Blood Centre of the Thai Red Cross in Bangkok in November 2023. Additionally, 222 leftover samples were obtained from individuals suspected of dengue virus (DENV) infection at Phonphisai hospital in Phonphisai district, Nong Khai Province, Thailand, between 2019 and 2020 (Fig. 1). Bangkok is the capital and most populous city of Thailand, with an estimated population of more than 11 million people as of 2024^17^. Phonphisai district is a rural area in the upper northeastern region of Thailand, with an estimated population of 71,864 people in 2024^18^.

Serum samples were sent to the Faculty of Medical Technology, Mahidol University in Nakhon Pathom, Thailand, in cold chain and were stored at − 20 °C prior to serological analyses. An anonymous data collection form was filled out by the National Blood Centre and included data on age, sex, province, district, and occupation. Data on age and sex were also gathered from individuals presenting dengue-like symptoms.

Ethical approval was obtained from the Mahidol University Central Institutional Review Board (COA No. MU-CIRB 2023/132.0409), the National Blood Center Thai Red Cross Society (COA No. NBC 15/2023), and Ethical Review Committee for Human Research Faculty of Public Health, Mahidol University (Protocol No. 88/2564). Written informed consent was obtained from adult participants, or parent/legal guardian in individuals under the age of 18 years, prior to enrolment.

### Human serological testing

A two-step method was used to detect IgG antibodies against SFTSV. First, serum samples were screened using a commercial anti-SFTSV IgG ELISA kit (Zhongshan Bio-tech Co., Ltd. Guangdong, China), based on the viral nucleoprotein, and performed according to the manufacturer’s instruction^19^. ELISA IgG was considered positive when the sample optical density (OD) was greater than or equal to the cutoff value (the average of OD value of negative control + 0.10). This commercial kit has been employed in other studies^19–22^, demonstrating its effectiveness in detecting SFTSV antibodies. Second, an in-house virus neutralization test (VNT) was performed for all samples with a positive ELISA result to detect SFTSV-specific neutralizing antibodies. One hundred TCID50 (tissue culture infectious dose) of the SFTSV SPL010A strain (provided by European Virus Archive Global (EVAg)) was mixed with equal volume of serially diluted serum (1:10 to 1:320) and incubated at 37 °C, 5% CO_2_ for 1 h. Subsequently, the mixture was transferred onto 96 well plate containing confluent Vero cells (ATCC CCL-81) and incubated at 37 °C, 5% CO_2_ for 1 h. Following incubation, serum + virus mix was removed, and cell monolayers were washed twice with Minimum Essential Medium (MEM). Finally, 200 µl of MEM/2,5% fetal bovine serum (FBS) was transferred on each well, and plates were incubated for 72 h at 37 °C, 5% CO_2_. As the SPL010A strain does not exhibit distinct cytopathic effect (CPE) on Vero cells, the endpoint assessment was conducted by quantifying the quantity of RNA copy numbers using qRT-PCR (Supplementary Table S1). At the end of the of the incubation; RNA was extracted from cell culture supernatants, and qRT-PCR was performed using sensitive and specific primers and probe (Supplementary Table S2) (These primers detect both Chinese and Japanese SFTSV and are capable of detecting viral RNA up to 1 TCDI50/reaction, as described^23^). The percentage reductions of viral copy number for each serum dilution were computed relative to the virus control, which included only the virus. Titer values were presented as the reciprocal of the serum dilution that reduced the RNA copy number in the virus control by at least 75%. A constructed control plasmid and a standard IVT-RNA with a known concentration (Supplementary Table S1) were used for qRT-PCR and VNT, respectively.

A sample was considered positive for SFTSV IgG antibodies when both, ELISA IgG and VNT were positive. For VNT tests, all experiments were performed in biosafety level 3 laboratory (BSL3) at the Unité des Virus Émergents, Marseille, France, according to standard BSL3 guidelines. All dilutions and liquid transfers were performed using an automated system (Epmotion 5075). Reactions were performed using Invitrogen SuperScript III reverse transcriptase (ThermoFisher Scientific, Massachusetts, USA) in a CFX96 thermal cycler (Bio-Rad, California, USA), according to the manufacturer’s instructions.

### Tick sample collection and ethical approval

In October, 2023, ticks were handpicked from 49 dogs in rural communities of Tha Takiap Subdistrict, Chachoengsao Province, Thailand, situated in the eastern part of the country (13° 22′ 53.3″ N 101° 42′ 04.0″ E) (Fig. 1), which had a population of 19,412 people by 2022^24^. The Tha Takiap Subdistrict is characterized by its hilly slopes, mountain ranges, and relatively intact rainforests. During the collection process, all dogs were captured and examined for ticks with the assistance of their owners. Ten ticks were randomly collected from each dog, in cases with fewer ticks, all available ticks were collected. After collection, the live tick samples were sent to the laboratory at the College of Allied Health Sciences, Suan Sunandha Rajabhat University’s Samut Songkhram Campus. The non-engorged ticks were euthanized by freezing at − 20 °C for 30 min. Subsequently, all ticks were washed in sterile physiological saline and preserved in RNAlater, stored in 1.5 ml microcentrifuge tubes at − 20 °C. Morphological identification of the ticks was performed using a taxonomic key^25^ under a Nikon SMZ 800 N stereo-microscope (Nikon Corp., Tokyo, Japan). Engorged female ticks were kept at room temperature for a week to encourage oviposition. After oviposition, these female ticks and their eggs were preserved and stored in microcentrifuge tubes at − 80 °C. The ticks were then grouped based on developmental stage (adult, nymph), sex (male, female), and the host animal from which they were collected. These categorized and labeled tubes were transported to the Faculty of Medical Technology at Mahidol University in Nakhon Pathom, Thailand, and stored at − 80 °C for further analysis.

The experimental protocol and tick sample collection were duly submitted for review and subsequently received approval from the Institutional Animal Care and Use Committee of Suan Sunandha Rajabhat University (No. IACUC 66–002/2023).

### DNA and RNA extraction

Microcentrifuge tubes containing pooled ticks were immersed in liquid nitrogen and crushed using a sterilized plastic pestle. After homogenization, the preparations were resuspended in 400–600 μl of sterile 1X phosphate-buffered saline (PBS) and stored at − 80 °C. Prior to extraction, the homogenates were centrifuged at 8000 rpm for 5 min at 4 °C, and the resulting supernatants were used to extract the total DNA/RNA using Nucleospin RNA virus extract kit (Macherey–Nagel, Germany), adding Proteinase K, following the manufacturer’s instructions, with a final elution volume of 80 μl. Extractions were subsequently stored at − 80 °C for further molecular analyses.

### PCR analyses

Taq-Man real-time polymerase chain reactions (qPCR or RT-qPCR) were employed to amplify pathogens, including SFTSV^23^, Crimean-Congo hemorrhagic fever (CCHF)^26^, *Coxiella* spp.^27^, *Bartonella* spp.^28^, and *Rickettsia* spp.^29^. Primers and probes (Supplementary Table S2), as well as bacteriophages MS2 and T4 (used as internal controls^30^), were provided in a lyophilized format^31^. Constructed plasmids were used as positive controls. All were kindly supplied by the European Virus Archive global (EVAg) and the Emerging Viruses Unit in Marseille, France. Reactions were performed using Invitrogen SuperScript III reverse transcriptase (ThermoFisher Scientific, Massachusetts, USA) in a CFX96 thermal cycler (Bio-Rad, California, USA), according to the manufacturer’s instructions. All methods were conducted in accordance with relevant guidelines and regulations.

### Statistical analysis

For the expected prevalence of 3.0% based on similar studies conducted in other countries, the required sample size was 1165 for the margin of error or absolute precision of ± 1 in estimating the prevalence with 95% confidence and considering the potential loss of 4.0%. With this sample size, the anticipated 95% confidence interval (CI) was 2.0–4.0%. The sample size was calculated using the Scalex SP calculator^32^.

## Results and discussion

The four human cases reported in Thailand identified in Bangkok and Chachoengsao Province between 2019 and 2020, indicate that SFTSV has been spreading in the country. Therefore, we examined the seroprevalence of anti-SFTSV IgG antibodies in 1162 human samples collected from 940 healthy blood donors in Bangkok with a median age of 41 years (range 17–68 years) and from 222 individuals with dengue-like symptoms in Nong Khai Province, with a median age of 15 years (range 8 months–67 years). The results of the initial ELISA screening for anti-SFTSV IgG antibodies revealed that 12 (1.1%) human samples were positive, with 5 from blood donors (3 company employees and 2 civil servants) and 7 from patients, with ELISA optical density ranging from 0.14 to 0.72 (mean OD = 0.26). The median age of the positive individuals was 40 years (range 16–57 years), with a prevalence of males 10/12 (83.3%). After confirmation by VNT, none of the samples tested positive, indicating the absence of neutralizing antibodies (Table 1).

**Table 1:** Human seroprevalence of severe fever with thrombocytopenia syndrome and molecular analysis of *Rhipicephalus sanguineus* ticks in Thailand.

Sample	Collection	Location	Province	SFTSV					
				ELISA		VNT		qPCR^a^	
				No. total	No. pos	No. total	No. pos	No. total	No. pos
Human sera	Blood donors	Thai Red Cross	Bangkok	940	5	5	0	NA	NA
Human sera	Dengue suspected	Phonphisai Hospital	Nong Khai	222	7	7	0	NA	NA
*Rh. sanguineus* ticks	Dogs	Tha Takiap Subdistrict	Chachoengsao	NA	NA	NA	NA	433 (50 pools)	0
*Rh. sanguineus eggs*	*Rh. sanguineus* ticks	Tha Takiap Subdistrict	Chachoengsao	NA	NA	NA	NA	12 pools	0

*ELISA* Enzyme-linked immunosorbent assay, *NA* Not applicable, *pos* Positive, *qPCR* Real-time polymerase chain reaction, *SFTSV* Severe fever with thrombocytopenia syndrome virus, *VNT* Virus neutralization test.

^a^Pathogens: SFTSV, Crimean-Congo hemorrhagic fever, *Coxiella* spp., *Bartonella* spp., and *Rickettsia* spp.

Seroprevalence studies have also been performed in other countries, including China (0.2–11.3%)^33,34^, Japan (0.0–4.2%)^34–39^, South Korea (1.9–7.7%)^40–44^, Pakistan (2.5%)^15^, and Vietnam (3.6%)^16^. The detection methods used were predominantly ELISA tests. Given that the seroprevalence rate in this current study was determined to be 1.1% using ELISA, this figure aligns with some studies from China, Japan, and South Korea. Further studies must be conducted to determine whether any viruses antigenically related to SFTSV are present in Thailand. For instance, Guertu virus (GTV), which was recently isolated from *D. nuttalli* ticks in China, is closely related to SFTSV and can also infect humans^45^.

Severe fever with thrombocytopenia syndrome in domesticated animals has recently gained public attention for its potential role as an infection source to humans. For instance, the first human case of SFTS in Thailand was a 70-year old woman suspected of having been infected by cats, as all seven of her cats died^13^. In Thailand, the presence of stray or neglected companion dogs, and the widespread popularity of dog ownership, pose an important source of infection^46^, as clinical signs of SFTSV-infected dogs are relatively mild, making the diagnosis difficult and barely attracting enough attention to their owners and veterinary professionals^37^. Moreover, a SFTSV seroprevalence study published in 2023 and conducted in dogs, demonstrated a total seroprevalence of 16.6% (ELISA and seroneutralization) in the provinces of Bangkok, Chachoengsao, Samut Prakan, Rayong, and Chonburi, suggesting that infection among the canine population may be similar to those in endemic regions of China^4^.

*Rh. sanguineus* tick infestation in dogs in Thailand is high (e.g. north-eastern 80.0%), which represents public health significance due to its greater likelihood of spreading pathogens. Since it requires different hosts for each blood meal, and in the era of globalization and climate changes, it has been suggested that ticks exposed to high temperatures attached more to humans, potentially increasing the risk of transmission of certain zoonotic pathogens^11^. Therefore, we collected 433 *Rhipicephalus sanguineus* sensu lato ticks from dogs in rural communities of Chachoengsao Province, Thailand, where one human case of SFTSV was identified in 2020^14^, and a seroprevalence of 11.1% was found in dogs in 2022 (confirmed by FRNT50)^4^. Two hundred fifty-three (58.4%) ticks were male, 55 (12.7%) were females and 125 (28.9%) engorged female ticks. Almost all were adults, except for four nymphs. In addition, 12 tubes of eggs were collected. All pool of ticks and eggs were tested for the detection of SFTSV (Table 1). However, none of the pools were positive for RNA detection. As *Haemaphysalis longicornis* serves as the primary vector, and considering that livestock are common hosts for this tick^47^, we also investigated the presence of ticks on 10 cattle and 15 goats in the same area of study. These animals are raised in the same household as dogs. However, no ticks were found on the livestock, probably due to the use of insecticides, since these animals are raised for sale.

*Rh. sanguineus* ticks have been identified as responsible for transmitting various well-known canine pathogens, including *Babesia vogeli*, *Babesia canis*, *Hepatozoon canis*, and *Mycoplasma haemocanis*, as well as pathogens capable of infecting both humans and dogs, such as *Ehrlichia canis*, *Anaplasma platys*, and several zoonotic Rickettsioses^11,48,49^. In this study, we tested less common but important pathogens that cause important health problems in humans, mainly transmitted by other tick species, but also found in *Rh. sanguineus* ticks, including CCHF, *Coxiella* spp., *Bartonella* spp.*,* and *Rickettsia* spp. However, we did not detect RNA or DNA from the collected tick samples.

This study has some limitations. Firstly, the seroprevalence study involved a disproportionately higher number of samples from urban areas compared to rural ones, with only a few samples from farmers. The sample size did not reflect the entire country’s population. Additionally, the study did not consider the influence of socioeconomic factors, outdoor activities, or history of tick-bites. Secondly, molecular identification was performed on a single tick and animal species and was confined to a specific area, potentially masking positive results. However, our study also has several strengths. Notably, this is the first seroprevalence study conducted on human samples in Thailand. The sample sizes are comparable to those of other countries and are appropriate for our study based on statistical formulas. Serum samples were collected in Bangkok, where most of the positive cases were identified. Collection from blood donors represents the healthy population and facilitates large-scale and cost-effective seroprevalence studies, allowing longitudinal trend analysis. In addition, serum samples were collected from a rural area bordering Laos, where the population may be more susceptible to tick-borne infections. The combination of ELISA and seroneutralization tests used in this study provides greater specificity, outperforming methodologies based solely on ELISA. Molecular identification of SFTSV in *Rhipicephalus sanguineus* ticks collected from dogs is significant due to the high infestation and the presence of human and dog infections found in the same province where the ticks were collected, potentially representing a source of infection. Although, no positive results were detected, this information is crucial for identifying potential increases in incidence or geographic range in the future. To achieve a more comprehensive understanding of the virus and the associated disease, it is important to conduct further studies across diverse geographic regions and on various tick species, including *Haemaphysalis longicornis*.

## Conclusion

Severe fever with thrombocytopenia syndrome virus poses an escalating global public health threat not only due to the segmented nature of its viral genome, which promotes frequent reassortment events, but also because of its increasing incidence, expanding geographical distribution, and high mortality rates. Individuals engaged in agriculture and forestry are considered at heightened risk of tick-borne infections. Moreover, close contact with domestic animals is also thought to be a significant risk factor for SFTSV transmission. To our knowledge, this is the first study of SFTSV seroprevalence in humans in Thailand. We demonstrate the current absence of neutralizing antibodies against SFTSV in the samples collected from Bangkok and Phon Phisai district (Nong Khai province). Additionally, we observed a considerable infestation of the tick species *Rhipicephalus sanguineus* in dogs in Chachoengsao, Thailand. Nonetheless, the presence of RNA or DNA of SFTSV, CCHF, *Coxiella* spp. *Bartonella* spp.*,* and *Rickettsia* spp. was not detected. Our findings highlight the importance of performing sustained surveillance to promptly detect emerging diseases in animals, vectors, and humans to better respond to health threats.

## Supplementary Information

Supplementary Information.

## Data Availability

The data that support the findings of the study are available within the article. Additional data are available upon request from the corresponding author.

## References

[1] Sharma, D. & Kamthania, M. A new emerging pandemic of severe fever with thrombocytopenia syndrome (SFTS). *VirusDisease* **32**, 220 (2021). 33942022 10.1007/s13337-021-00656-9 PMC8082055

[2] Casel, M. A., Park, S. J. & Choi, Y. K. Severe fever with thrombocytopenia syndrome virus: Emerging novel phlebovirus and their control strategy. *Exp. Mol. Med.* **53**, 713–722 (2021). 33953322 10.1038/s12276-021-00610-1 PMC8178303

[3] Seo, J.-W., Kim, D., Yun, N. & Kim, D.-M. Clinical update of severe fever with thrombocytopenia syndrome. *Viruses* **13**, 1213 (2021). 34201811 10.3390/v13071213 PMC8310018

[4] Ishijima, K. *et al.* High seroprevalence of severe fever with thrombocytopenia syndrome virus infection among the dog population in Thailand. *Viruses* **15**, 2403 (2023). 38140644 10.3390/v15122403 PMC10747823

[5] Yang, T., Huang, H., Jiang, L. & Li, J. Overview of the immunological mechanism underlying severe fever with thrombocytopenia syndrome (review). *Int. J. Mol. Med.* **50**, 118 (2022). 35856413 10.3892/ijmm.2022.5174 PMC9333902

[6] Sul, H. *et al.* Development of a scoring system to differentiate severe fever with thrombocytopenia syndrome from scrub typhus. *Viruses* **14**, 1093 (2022). 35632834 10.3390/v14051093 PMC9143636

[7] Han, X.-H. *et al.* Identification of severe fever with thrombocytopenia syndrome virus genotypes in patients and ticks in Liaoning Province, China. *Parasites Vectors* **15**, 120 (2022). 35379310 10.1186/s13071-022-05237-3 PMC8981814

[8] Xing, Y. *et al.* Epidemiological investigation of predominance tick and the infectious status of severe fever thrombocytopenia syndrome virus in Penglai and Laizhou counties, Shandong province. *Zhonghua Yu Fang Yi Xue Za Zhi* **49**, 993–997 (2015). 26833011

[9] Hu, Y.-Y. *et al.* Role of three tick species in the maintenance and transmission of Severe Fever with Thrombocytopenia Syndrome Virus. *PLOS Negl. Trop. Dis.* **14**, e0008368 (2020). 32520966 10.1371/journal.pntd.0008368 PMC7307786

[10] Yuan, C. *et al.* Infection and transovarial transmission of severe fever with thrombocytopenia syndrome virus in *Rhipicephalus sanguineus* in Hainan Island, China. *Integr. Zool.* **18**, 1009–1013 (2023). 36905201 10.1111/1749-4877.12716

[11] Dantas-Torres, F. Biology and ecology of the brown dog tick, Rhipicephalus sanguineus. *Parasites & Vectors* **3**, 26 (2010). 20377860 10.1186/1756-3305-3-26 PMC2857863

[12] Yu, X.-J. *et al.* Fever with thrombocytopenia associated with a novel bunyavirus in China. *N Engl J Med* **364**, 1523–1532 (2011). 21410387 10.1056/NEJMoa1010095 PMC3113718

[13] Ongkittikul, S., Watanawong, R. & Rompho, P. Severe fever with thrombocytopenia syndrome virus: The first case report in Thailand. *Bangkok Med. J.* **16**, 204–204 (2020).

[14] Rattanakomol, P. *et al.* Severe fever with thrombocytopenia syndrome virus infection, Thailand, 2019–2020. *Emerg. Infect. Dis.* **28**, 2572–2574 (2022). 36418010 10.3201/eid2812.221183 PMC9707585

[15] Zohaib, A. *et al.* Serologic evidence of severe fever with thrombocytopenia syndrome virus and related viruses in Pakistan. *Emerg. Infect. Dis. J. CDC*10.3201/eid2607.190611 (2020). 10.3201/eid2607.190611 PMC7323538 32568060

[16] Tran, X. C. *et al.* Serological evidence of severe fever with thrombocytopenia syndrome virus and IgM positivity were identified in healthy residents in Vietnam. *Viruses* **14**, 2280 (2022). 36298836 10.3390/v14102280 PMC9607213

[17] Bangkok Population 2024. https://worldpopulationreview.com/world-cities/bangkok-population.

[18] 3 หมอรู้จักคุณ :: กรมสนับสนุนบริการสุขภาพ. https://3doctor.hss.moph.go.th/main/rp_tambon?region=8&prov=NDM=&provn=4Lir4LiZ4Lit4LiH4LiE4Liy4Lii&ampid=4305&ampn=4LmC4Lie4LiZ4Lie4Li04Liq4Lix4Lii.

[19] Huang, X. *et al.* Estimation of the incidence of severe fever with thrombocytopenia syndrome in high endemic areas in China: An inpatient-based retrospective study. *BMC Infect. Dis.* **18**, 66 (2018). 29402229 10.1186/s12879-018-2970-7 PMC5800001

[20] Direct transmission of severe fever with thrombocytopenia syndrome virus from farm-raised fur animals to workers in Weihai, China | Virology Journal | Full Text. 10.1186/s12985-024-02387-x. 10.1186/s12985-024-02387-x PMC11100147 38760812

[21] Liang, S., Li, Z., Zhang, N., Wang, X. & Hu, J. Detection of severe fever with thrombocytopenia syndrome virus RNA and total antibodies in wild animals, Jiangsu, China, 2014–2019. *J. Public Health Emerg.* **6**, (2022).

[22] Quan, C. *et al.* SFTSV infection is associated with transient overproliferation of monoclonal lambda-type plasma cells. *iScience* **26**, 106799 (2023). 37250798 10.1016/j.isci.2023.106799 PMC10212991

[23] Yoshikawa, T. *et al.* Sensitive and specific PCR systems for detection of both Chinese and Japanese severe fever with thrombocytopenia syndrome virus strains and prediction of patient survival based on viral load. *J. Clin. Microbiol.* **52**, 3325–3333 (2014). 24989600 10.1128/JCM.00742-14 PMC4313158

[24] องค์การบริหารส่วนตำบลท่าตะเกียบ. https://www.thatakieb.go.th/event.php.

[25] Farid, H. A. Morphological keys for the separation of the *Rhipicephalus sanguineus* group of ticks (Acarina:Ixodidae) in Egypt. *J. Egypt Soc. Parasitol.* **26**, 453–460 (1996). 8754653

[26] Wölfel, R. *et al.* Virus detection and monitoring of viral load in Crimean-Congo hemorrhagic fever virus patients. *Emerg. Infect. Dis.* **13**, 1097–1100 (2007). 18214191 10.3201/eid1307.070068 PMC2878241

[27] Mediannikov, O. *et al.**Coxiella burnetii* in humans and ticks in rural Senegal. *PLoS Negl Trop Dis* **4**, e654 (2010). 20386603 10.1371/journal.pntd.0000654 PMC2850317

[28] Socolovschi, C., Kernif, T., Raoult, D. & Parola, P. Borrelia, Rickettsia, and Ehrlichia species in bat ticks, France, 2010. *Emerg. Infect. Dis.* **18**, 1966–1975 (2012). 23171714 10.3201/eid1812.111237 PMC3557878

[29] Wright, C. L. *et al.* Rickettsia parkeri in gulf coast ticks, southeastern Virginia, USA. *Emerg. Infect. Dis.* **17**, 896–898 (2011). 21529406 10.3201/eid1705.101836 PMC3321792

[30] Ninove, L. *et al.* RNA and DNA bacteriophages as molecular diagnosis controls in clinical virology: A comprehensive study of more than 45,000 routine PCR tests. *PLoS One* **6**, e16142 (2011). 21347398 10.1371/journal.pone.0016142 PMC3036576

[31] Thirion, L. *et al.* Lyophilized matrix containing ready-to-use primers and probe solution for standardization of real-time PCR and RT-qPCR diagnostics in virology. *Viruses* **12**, 159 (2020). 32019076 10.3390/v12020159 PMC7077261

[32] Naing, L., Nordin, R. B., Abdul Rahman, H. & Naing, Y. T. Sample size calculation for prevalence studies using Scalex and ScalaR calculators. *BMC Med. Res. Methodol.* **22**, 209 (2022). 35907796 10.1186/s12874-022-01694-7 PMC9338613

[33] Li, P. *et al.* Seroprevalence of severe fever with thrombocytopenia syndrome virus in China: A systematic review and meta-analysis. *PLoS One* **12**, e0175592 (2017). 28399161 10.1371/journal.pone.0175592 PMC5388504

[34] Huang, X. Y. *et al.* Severe fever with thrombocytopenia syndrome virus: A systematic review and meta-analysis of transmission mode. *Epidemiol. Infect.* **148**, e239 (2020). 32993819 10.1017/S0950268820002290 PMC7584033

[35] Kimura, T. *et al.* Seroprevalence of severe fever with thrombocytopenia syndrome (SFTS) virus antibodies in humans and animals in Ehime prefecture, Japan, an endemic region of SFTS. *J. Infect. Chemother.* **24**, 802–806 (2018). 30017796 10.1016/j.jiac.2018.06.007

[36] Gokuden, M. *et al.* Low seroprevalence of severe fever with thrombocytopenia syndrome virus antibodies in individuals living in an endemic area in Japan. *Jpn. J. Infect. Dis.* **71**, 225–228 (2018). 29709983 10.7883/yoken.JJID.2017.497

[37] Kirino, Y. *et al.* Retrospective study on the possibility of an SFTS outbreak associated with undiagnosed febrile illness in veterinary professionals and a family with sick dogs in 2003. *J. Infect. Chemother.* **28**, 753–756 (2022). 35219579 10.1016/j.jiac.2022.02.011

[38] Ando, T. *et al.* Severe fever with thrombocytopenia syndrome in cats and its prevalence among veterinarian staff members in Nagasaki, Japan. *Viruses* **13**, 1142 (2021). 34198717 10.3390/v13061142 PMC8232257

[39] Matsumoto, C. *et al.* Investigation of antibody to severe fever with thrombocytopenia syndrome virus (SFTSV) in blood samples donated in a SFTS-endemic area in Japan. *Vox Sang.* **113**, 297–299 (2018). 29359332 10.1111/vox.12629

[40] Han, M. A. *et al.* Seroprevalence of severe fever with thrombocytopenia syndrome virus antibodies in rural areas, South Korea. *Emerg. Infect. Dis.* **24**, 872–874 (2018). 29664384 10.3201/eid2405.152104 PMC5938763

[41] Kim, C.-M. *et al.* Seroprevalence of severe fever with thrombocytopenia syndrome using specimens from the Korea National Health & Nutrition Examination Survey. *PLOS Negl. Trop. Dis.* **17**, e0011097 (2023). 36947741 10.1371/journal.pntd.0011097 PMC10032665

[42] Kim-Jeon, M. D. *et al.* Seroprevalence of severe fever with thrombocytopenia syndrome virus in Mui Island, Rural Area, Incheon, South Korea. *J. Bacteriol. Virol.* **52**, 64–71 (2022).

[43] Yoo, J. R. *et al.* Seroprevalence of severe fever with thrombocytopenia syndrome in the agricultural population of Jeju Island, Korea, 2015–2017. *Infect. Chemother.* **51**, 337–344 (2019). 31668024 10.3947/ic.2019.51.4.337 PMC6940373

[44] Kim, K.-H., Ko, M. K., Kim, N., Kim, H. H. & Yi, J. Seroprevalence of severe fever with thrombocytopenia syndrome in Southeastern Korea, 2015. *J. Korean Med. Sci.* **32**, 29–32 (2017). 27914128 10.3346/jkms.2017.32.1.29 PMC5143294

[45] Shen, S. *et al.* A novel tick-borne phlebovirus, closely related to severe fever with thrombocytopenia syndrome virus and Heartland virus, is a potential pathogen. *Emerg. Microbes Infect.* **7**, 95 (2018). 29802259 10.1038/s41426-018-0093-2 PMC5970217

[46] Colella, V. *et al.* Zoonotic vectorborne pathogens and ectoparasites of dogs and cats in Eastern and Southeast Asia. *Emerg. Infect. Dis. J. CDC*10.3201/eid2606.191832 (2020). 10.3201/eid2606.191832 PMC7258489 32441628

[47] Zhang, X. *et al.* Rapid spread of severe fever with thrombocytopenia syndrome virus by parthenogenetic Asian longhorned ticks. *Emerg. Infect. Dis. J. CDC*10.3201/eid2802.211532 (2022). 10.3201/eid2802.211532 PMC8798674 35075994

[48] Do, T. *et al.* Molecular detection of tick-borne pathogens in stray dogs and *Rhipicephalussanguineus sensu* lato ticks from Bangkok, Thailand. *Pathogens* **10**, 561 (2021). 34066526 10.3390/pathogens10050561 PMC8148546

[49] Galay, R. L. *et al.* Molecular detection of tick-borne pathogens in canine population and Rhipicephalus sanguineus (sensu lato) ticks from southern Metro Manila and Laguna, Philippines. *Parasit Vectors* **11**, 643 (2018). 30558678 10.1186/s13071-018-3192-y PMC6296069

