# Author Correction: Seroprevalence study in humans and molecular detection in *Rhipicephalus sanguineus* ticks of severe fever with thrombocytopenia syndrome virus in Thailand

**DOI:** 10.1038/s41598-025-89292-7

**Published:** 2025-02-13

**Authors:** Paola Mariela Saba Villarroel, Tanawat Chaiphongpachara, Elif Nurtop, Sedthapong Laojun, Tassanee Pangpoo-nga, Thanaphon Songhong, Dolruethai Supungul, Cécile Baronti, Laurence Thirion, Pornsawan Leaungwutiwong, Xavier de Lamballerie, Dorothée Missé, Sineewanlaya Wichit

**Affiliations:** 1 Department of Clinical Microbiology and Applied Technology, Faculty of Medical Technology, Mahidol University, Nakhon Pathom, Thailand; 2 Viral Vector Joint Unit and Joint Laboratory, Mahidol University, Nakhon Pathom, Thailand; 3 Department of Public Health and Health Promotion, College of Allied Health Sciences, Suan Sunandha Rajabhat University, Samut Songkhram, Thailand; 4 Unité des Virus Émergents, (UVE: Aix-Marseille Univ-IRD190- Inserm 1207), Marseille, France; 5 Phon Phisai Hospital, Phon Phisai District, Nong Khai Thailand; 6 Department of Microbiology and Immunology, Faculty of Tropical Medicine, Mahidol University, Bangkok, Thailand; 7 MIVEGEC, CNRS, IRD, Univ. Montpellier, Montpellier, France

Correction to: *Scientific Reports* 10.1038/s41598-024-64242-x, published online 11 June 2024

The original version of this Article contained an error in the Ethics number in the Materials and Methods section, under the subheading ‘Human sample collection and ethical approval’,

“Ethical approval was obtained from the Mahidol University Central Institutional Review Board (COA No. MU-CIRB 2023/167.2505).”

now reads:

“Ethical approval was obtained from the Mahidol University Central Institutional Review Board (COA No. MU-CIRB 2023/132.0409).”

The original Article has been corrected.

