# Supplementary material for: Seroprevalence study in humans and molecular detection in Rhipicephalus sanguineus ticks of severe fever with thrombocytopenia syndrome virus in Thailand
Source: Sci Rep. 2024 Jun 11;14:13397. doi: 10.1038/s41598-024-64242-x (PMC11167008; doi:10.1038/s41598-024-64242-x)
Supplement: Supplementary file 1 — Supplementary Information. [file 41598_2024_64242_MOESM1_ESM.pdf]

Seroprevalence study in humans and molecular detection in *Rhipicephalus sanguineus* ticks of severe fever with thrombocytopenia syndrome virus in Thailand

Paola Mariela Saba Villarroel, Tanawat Chaiphongpachara, Elif Nurtop, Sedthapong Laojun, Tassanee Pangpoo-nga, Thanaphon Songhong, Dolruethai Supungul, Cécile Baronti, Laurence Thirion, Pornsawan Leaungwutiwong, Xavier de Lamballerie, Dorothée Missé, Sineewanlaya Wichit

Supplementary table 1. In- house virus neutralization test (VNT) results

Sample (dilution)	Cq	SQ
29 (1/20)	31.95	5353
29 (1/40)	32.46	3923
29 (1/80)	29.54	22663
29 (1/160)	30.53	12491
29 (1/320)	29.32	25775
29 (1/640)	29.90	18245
72 (1/20)	28.70	37544
72 (1/40)	29.26	26815
72 (1/80)	29.47	23600
72 (1/160)	31.73	6075
72 (1/320)	28.48	42771
72 (1/640)	29.95	17708
78 (1/20)	29.37	25047
78 (1/40)	29.70	20642
78 (1/80)	29.34	25565
78 (1/160)	29.42	24374
78 (1/320)	31.34	7699
78 (1/640)	31.11	8822
126 (1/20)	32.38	4131
126 (1/40)	30.02	17025
126 (1/80)	28.95	32341
126 (1/160)	29.16	28528
126 (1/320)	31.46	7159

Sample	Cq	SQ
**Standard**	19.73	6580000
**Standard**	23.73	658000
**Standard**	27.78	65800
**Standard**	31.49	6580
**Standard**	35.38	658

126 (1/640)	29.30	26241
128 (1/20)	30.32	14226
128 (1/40)	29.01	31181
128 (1/80)	28.38	45558
128 (1/160)	32.11	4845
128 (1/320)	28.37	45777
128 (1/640)	29.90	18245
180 (1/20)	28.08	54489
180 (1/40)	29.54	22664
180 (1/80)	27.93	59579
180 (1/160)	30.16	15593
180 (1/320)	31.43	7290
180 (1/640)	28.75	36304
193 (1/20)	28.58	40228
193 (1/40)	28.29	47952
193 (1/80)	28.58	40403
193 (1/160)	31.69	6227
193 (1/320)	31.98	5249
193 (1/640)	29.01	31181
242 (1/20)	29.42	24374
242 (1/40)	29.47	23600
242 (1/80)	28.48	42771
242 (1/160)	30.54	12416
242 (1/320)	30.53	12504
242 (1/640)	29.96	17610
259 (1/20)	29.26	26815
259 (1/40)	29.27	26702
259 (1/80)	30.02	17025
259 (1/160)	29.11	23856
259 (1/320)	29.70	20642
259 (1/640)	30.10	12990
571 (1/20)	29.54	22664
571 (1/40)	31.69	6227
571 (1/80)	31.46	7159
571 (1/160)	30.53	12491
571 (1/320)	32.46	3923
571 (1/640)	29.27	26702
594 (1/20)	31.11	8822
594 (1/40)	31.98	5249
594 (1/80)	30.16	15593
594 (1/160)	29.16	28528

594 (1/320)	29.34	25565
594 (1/640)	31.95	5353
719 (1/20)	31.43	7290
719 (1/40)	32.46	3923
719 (1/80)	31.34	7699
719 (1/160)	31.73	6075
719 (1/320)	29.96	17610
719 (1/640)	30.32	14226
Virus	29.01	31181
Virus	30.53	12503
Virus	30.21	15135
Virus	29.96	17610
Virus	30.54	12416
Virus	29.27	26701
Neg Ctrl		

Supplementary table S2. Primers and probes used for pathogens molecular detection

Pathogen	Oligo	5'-3' sequence	Reference
Bartonella spp.	PF	GAT GCC GGG GAA GGT TTT C	Socolovschi C. et al., 2012 [1]
	PR	GCC TGG GAG GAC TTG AAC CT	
	Probe	FAM-GCG CGC GCT TGA TAA GCG TG-TAMRA	
Rickettsia spp.	PF	GGG CGG TAT GAA YAA ACA AG	Wright C. et al., 2011[2]
	PR	CCT ACA CCT ACT CCV ACA AG	
	Probe	FAM-CCG AAT TGA GAA CCA AGT AAT GC-TAMRA	
Coxiella spp.	PF	CAA GAA ACG TAT CGC TGT GGC	Mediannikov O. et al., 2010[3]
	PR	CAC AGA GCC ACC GTA TGA ATC	
	Probe	FAM-CCG AGT TCG AAA CAA TGA GGG CTG-TAMRA	
CCHF	PF	CAA GGG GTA CCA AGA AAA TGA AGA AGG C	Wölfel R. et al., 2007[4]
	PR	GCC ACA GGG ATT GTT CCA AAG CAG AC	
	Probe	FAM-ATC TAC ATG CAC CCT GCT GTG TTG ACA-TAMRA	
SFTSV	PF	TGT CAG AGT GGT CCA GGA TT	Yoshikawa T., et al. 2014[5]
	PR	ACC TGT CTC CTT CAG CTT CT	
	Probe	FAM-TGG AGT TTG GTG AGC AGC-BHQ1	

Abbreviations: CCHF: Crimean-Congo hemorrhagic fever; PF: Primer forward; PR: Primer reverse; P: Probe; SFTSV: Severe fever with thrombocytopenia syndrome virus

References:

1. Socolovschi, C., Kernif, T., Raoult, D. & Parola, P. Borrelia, Rickettsia, and Ehrlichia Species in Bat Ticks, France, 2010. *Emerg Infect Dis* **18**, 1966–1975 (2012).
2. Wright, C. L. *et al.* Rickettsia parkeri in gulf coast ticks, southeastern Virginia, USA. *Emerg Infect Dis* **17**, 896–898 (2011).
3. Mediannikov, O. *et al.* Coxiella burnetii in Humans and Ticks in Rural Senegal. *PLoS Negl Trop Dis* **4**, e654 (2010).
4. Wölfel, R. *et al.* Virus Detection and Monitoring of Viral Load in Crimean-Congo Hemorrhagic Fever Virus Patients. *Emerg Infect Dis* **13**, 1097–1100 (2007).
5. Yoshikawa, T. *et al.* Sensitive and Specific PCR Systems for Detection of Both Chinese and Japanese Severe Fever with Thrombocytopenia Syndrome Virus Strains and Prediction of Patient Survival Based on Viral Load. *Journal of Clinical Microbiology* **52**, 3325–3333 (2014).
